# Nonlinear Changes in Dryland Vegetation Greenness over East Inner Mongolia, China, in Recent Years from Satellite Time Series

**DOI:** 10.3390/s20143839

**Published:** 2020-07-09

**Authors:** Chao Ding, Wenjiang Huang, Yao Li, Shuang Zhao, Fang Huang

**Affiliations:** 1 Key Laboratory of Digital Earth Science, Aerospace Information Research Institute, Chinese Academy of Sciences, Beijing 100094, China; dingchao@aircas.ac.cn; 2 Zachry Department of Civil and Environmental Engineering, Texas A&M University, College Station, TX 77843, USA; liyao@tamu.edu; 3 School of Geology and Geometics, Tianjin Chengjian University, Tianjin 300384, China; zhaoshuang@tcu.edu.cn; 4 School of Geographical Sciences, Northeast Normal University, Changchun 130024, China; huangf835@nenu.edu.cn

**Keywords:** land desertification, remote sensing, enhanced vegetation index, leaf area index, vegetation trend, nonlinear changes, East Inner Mongolia

## Abstract

Knowledge of the dynamics of dryland vegetation in recent years is essential for combating desertification. Here, we aimed to characterize nonlinear changes in dryland vegetation greenness over East Inner Mongolia, an ecotone of forest–grassland–cropland in northern China, with time series of Moderate-resolution Imaging Spectroradiometer (MODIS) enhanced vegetation index (EVI) and GEOV2 leaf area index (LAI) values during 2000 to 2016. Changes in the growing season EVI and LAI were detected with the polynomial change fitting method. This method characterizes nonlinear changes in time series by polynomial fitting with the highest polynomial order of three, and simultaneously provides an estimation of monotonic trends over the time series by linear fitting. The relative contribution of climatic factors (precipitation and temperature) to changes in the EVI and LAI were analyzed using linear regression. In general, we observed similar patterns of change in the EVI and LAI. Nonlinear changes in the EVI were detected for about 21% of the region, and for the LAI, the percentage of nonlinear changes was about 16%. The major types of nonlinear changes include decrease–increase, decrease–increase–decrease, and increase–decrease–increase changes. For the overall monotonic trends, very small percentages of decrease (less than 1%) and widespread increases in the EVI and LAI were detected. Furthermore, large areas where the effects of climate variation on vegetation changes were not significant were observed for all major types of change in the grasslands and rainfed croplands. Changes with an increase–decrease–increase process had large percentages of non-significant effects of climate. The further analysis of increase–decrease–increase changes in different regions suggest that the increasing phases were likely to be mainly driven by human activities, and droughts induced the decreasing phase. In particular, some increase–decrease changes were observed around the large patch of bare areas. This may be an early signal of degradation, to which more attention needs to be paid to combat desertification.

## 1. Introduction

Diverse types of change in ecosystem parameters across global terrestrial ecosystems have been observed by satellite time series with different nonlinear change detection algorithms, e.g., [1,2,3,4,5]. These studies have revealed more detailed spatiotemporal patterns in vegetation changes than linear trend analysis and demonstrated the benefits of analyzing nonlinear changes for the elucidation of the complex interactions between vegetation and the environment over recent decades [1,2,3,4,5]. Importantly, there are large areas showing trend reversal, from vegetation greening to browning, due to various factors, which highlights the potential risk of land degradation in these areas [1,4].

East Inner Mongolia—an ecotone of forest, grassland, and cropland in northern China, East Asia—has been affected by severe land desertification for decades, mainly due to overgrazing and grassland reclamation [6,7,8]. Recent studies using satellite-derived time series have observed some greening areas in northern China, e.g., [9,10,11,12,13,14]. Some of the greening trends were attributed to afforestation efforts to combat desertification under several ecological restoration projects [9,10,13]. The greening process may be more evident after 2000 because most of these projects were implemented after that year [13,14]. Irrigation and fertilization may be other primary drivers of vegetation greening in agricultural areas [9,15,16,17].

In addition to linear trend analysis, the analysis of nonlinear changes should be helpful for providing deeper insights into land degradation and recovery processes in this ecologically vulnerable area [14]. The risk of land degradation still exists in East Inner Mongolia mainly due to inherent ecological vulnerability and climate change [18,19,20]. A major environmental challenge to human efforts to restore ecosystems in this region is drought [21,22]. The negative impacts of droughts on vegetation recovery in northern China have been reported [23,24,25]. In particular, they may cause the mortality of planted trees [23,26], which has been identified as an important issue in China’s forest–grassland ecotone [23]. Whether dryland ecosystems across this region have exhibited a decline in vegetation greenness in the most recent years after vegetation greening is therefore especially important. Other types of nonlinear changes may also be informative for depicting specific processes. Most of the recent nonlinear analyses of the vegetation greenness covering the region were based on AVHRR time series (since 1982) with an 8 km spatial resolution, e.g., [14,27]. Therefore, these may not depict nonlinear changes over the past two decades in enough detail, which is key for capturing the most recent nonlinear vegetation changes for East Inner Mongolia. Qiu et al. [28] proposed a framework for characterizing nonlinear vegetation changes and applied it in northern China using MODIS data for the period 2001–2015. However, this framework does not contain the types of trend reversal. Ma et al. [29] analyzed changes in gross primary productivity using the Breaks for Additive Seasonal and Trend (BFAST, [30]) across China during 2000 to 2016 and revealed some nonlinear changes in East Inner Mongolia. This analysis considered two types of trend reversal but did not consider change types of multiple reversal—for example, increase–decrease–increase changes.

In this investigation, we aimed to improve the understanding of land desertification and vegetation changes over East Inner Mongolia in recent years based on the nonlinear analysis of satellite-derived vegetation greenness. We detected changes in vegetation greenness using the MODIS enhanced vegetation index (EVI, [31]) and GEOV2 LAI during 2000 to 2016 by the polynomial change fitting method [32]. Relative effects of climate and non-climate factors on changes in the EVI and LAI were evaluated by linear regression and the residual trend analysis [33]. 

## 2. Materials and Methods

### 2.1. Study Area

East Inner Mongolia is in the transition zone of the semi-arid and sub-humid temperate continental monsoon climates in northern China. The spatial pattern of precipitation is heterogeneous across the region, with the average annual precipitation for the period of 2000-2016 ranging from approximately 200 to 550 mm (calculated with precipitation data in Section 2.2.4) and large interannual and intra-annual dynamics. Most precipitation occurs in summer (June to August), and snowfall generally occurs from early November to late March.

**Figure 1:**
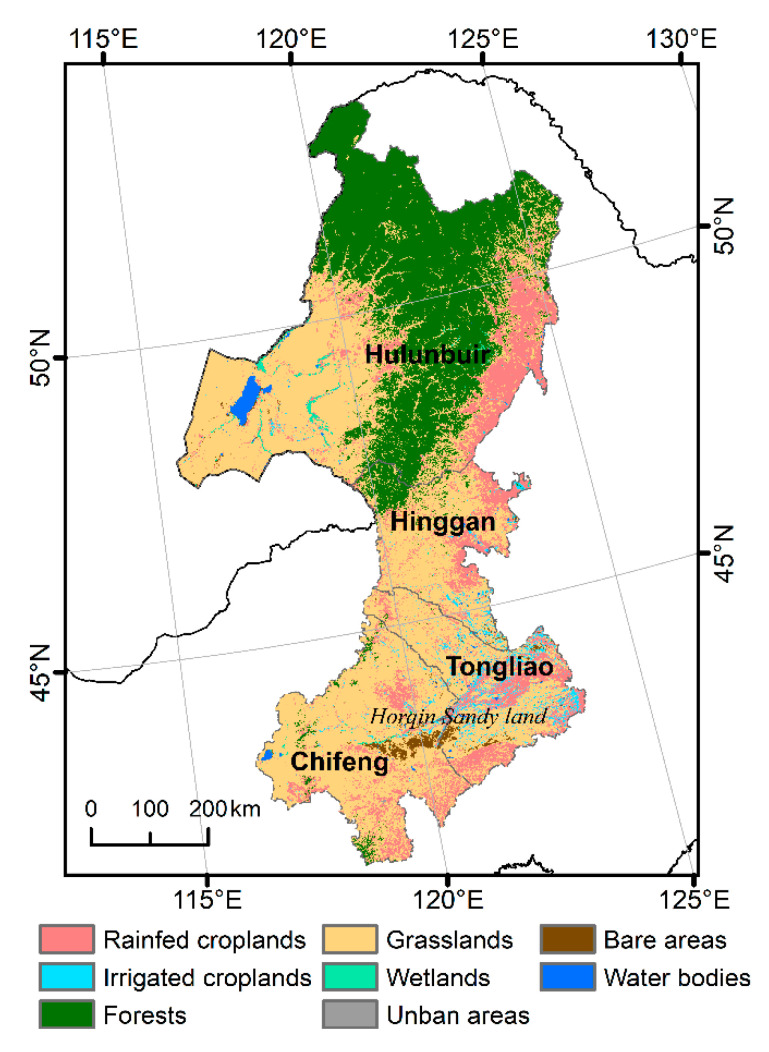
Land cover (reclassified ESA CCI) of East Inner Mongolia, China.

This ecotone consists of various landscapes (Figure 1). The northern areas are dominated by deciduous broad-leaved and deciduous-coniferous forests. Grasslands and croplands dominate other areas. Land desertification is widespread over this region [8,34], especially in Chifeng, Tongliao, and east Hulunbuir.

### 2.2. Data

#### 2.2.1. MODIS Enhanced Vegetation Index (EVI) Product

The dynamics of vegetation greenness were analyzed using MODIS/Terra 16-day composite EVI data (MOD13A2 Collection 6) with a 1 km resolution. The MOD13A2 data for 2000-2016 were collected from the Level-1 and Atmosphere Archive & Distribution System (LAADS) Distributed Active Archive Center (DAAC) (https://ladsweb.modaps.eosdis.nasa.gov/). Because the study area may be covered by snow in winter, we only used the data during the growing season (mid-April to mid-October). We replaced observations identified as cloudy in the quality flags by the corresponding multi-year average values [35]. We then calculated the growing season mean EVI values for each year.

#### 2.2.2. GEOV2 LAI Product

We also used another greenness-related land surface variable, the LAI, to corroborate the analysis of the change in the MODIS EVI, to reduce uncertainty [36,37,38]. The 1 km 10-day composite GEOV2 LAI data from 2000 to 2016 were acquired from the Copernicus Global Land Service (CGLS) (https://land.copernicus.eu/global/). The GEOV2 LAI is estimated using a neural network algorithm with SPOT/VEGETATION and PROBA-V reflectance as inputs [39]. This time series product is gap filled and temporally smoothed. We used the growing season mean LAI time series for the analysis of change.

#### 2.2.3. ESA CCI Land Cover Product

We obtained the 300 m CCI land cover product for 2000 from http://maps.elie.ucl.ac.be/CCI/viewer/. In this product, land cover types is classified using the United Nations (UN) Food and Agriculture Organization (FAO) classification system [40], and we combined the original land cover types at a broader level for further analysis using the reclassification rule presented in [41]. This rule holds for rainfed and irrigated croplands and is therefore helpful for analyzing the drivers of changes in greenness in dryland agricultural areas. We then aggregated the reclassified land cover map to a 1 km resolution (Figure 1). Change detection was not performed for pixels classified as urban areas, water bodies, and wetlands.

#### 2.2.4. Meteorological Data

The precipitation and air temperature data from the China Meteorological Forcing Dataset for 2000–2016 were obtained from http://www.tpedatabase.cn/. This reanalyzed dataset provides 3-h near-surface meteorological data at a 0.1° spatial resolution [42,43]. We used the total precipitation and mean air temperature from April to October for further analysis. These data were resampled to a 1 km resolution by nearest neighbor interpolation [44,45].

### 2.3. Methods

#### 2.3.1. Detection of Nonlinear Changes in EVI and LAI

We focused on nonlinear changes in vegetation greenness to characterize more detailed processes of land degradation and recovery. Nonlinear changes in the growing season mean EVI and LAI time series (2000–2016) were detected using the polynomial change fitting method developed in [32]. This method characterizes the nonlinear process in time series by polynomial fitting with the highest polynomial order of three and simultaneously provides an estimation of the monotonic trend over the time series by linear trend fitting. Table 1 presents the types of change detected with this method [3,32]. Each nonlinear change contains three sub-types according to the overall monotonic trend. For example, cubic change of the increase–decrease–increase type (CIDI) can be divided into a CIDI with an increasing overall trend (CIDI_I), a decreasing trend (CIDI_D), and a non-significant overall—i.e., “concealed”—trend (CIDI_C) [21].

The procedure of identifying changes in the EVI and LAI time series for a pixel is described below:

Step 1: First, a cubic function was fitted to the time series. Cubic change was identified if the cubic fitting met the following conditions: (1) the coefficient of the cubic fitting was statistically significant, and (2) the two local extreme points of the cubic function occurred during the study period. It is worth noting that a cubic function can also be monotonic, but in this method, only the non-monotonic form that has two extreme points was selected.
Step 2: If the time series were not identified as exhibiting a cubic change, the presence of a quadratic change was then tested. Similarly, a quadratic change was identified using the following conditions: (1) the coefficient of the quadratic fitting was statistically significant, and (2) the extreme point of the quadratic function occurred during the study period.
Step 3: Linear change fitting was performed to examine the overall monotonic trend. If the time series showed a cubic or quadratic change, such change was then divided into three sub-types based on the overall trend detected by the linear fitting as described above. Otherwise, it was judged that there was no significant nonlinear change in the time series, and the change was identified as linear or not significant.

#### 2.3.2. Regression Analysis of Vegetation Greenness and Climate and Residual Trend Analysis

We used multiple linear regression between time series of vegetation greenness and climate to evaluate the relative impacts of climate on changes in vegetation greenness [45]. The climate factors used include the total precipitation and mean temperature of the growing season. For areas with non-significant regression, changes in vegetation greenness can be attributed to non-climate factors [45,46]. For areas with statistically significant regression, we also performed residual trend analysis to assess whether there were negative trends in the EVI or LAI induced by non-climate factors [33]. Residual trend analysis is developed on the basis that naturally, dryland vegetation productivity is predominantly affected by climate variation, especially regarding precipitation [33]. Therefore, to avoid ambiguous results, we only applied this method to grasslands and rainfed croplands, which are typical dryland ecosystems expected to be sensitive to interannual climate variation [33,47]. Irrigated croplands were excluded from the analysis [48]. Forests were also not considered because the relationship between greenness and productivity may not be strong [49]. Please note that if the coefficient of the regression for precipitation was negative, it was also identified as non-significant regression [48,49], because dryland vegetation greenness is generally positively correlated with precipitation. If the regression was significant (*p* < 0.05), we calculated the residual of the regression and then analyzed trends in the residual time series by linear trend fitting. Specific analysis was performed for some areas showing QID changes or nonlinear changes that were not heavily influenced by climate variation [2,47].

## 3. Results

### 3.1. Changes in MODIS EVI and GEOV2 LAI

**Figure 2:**
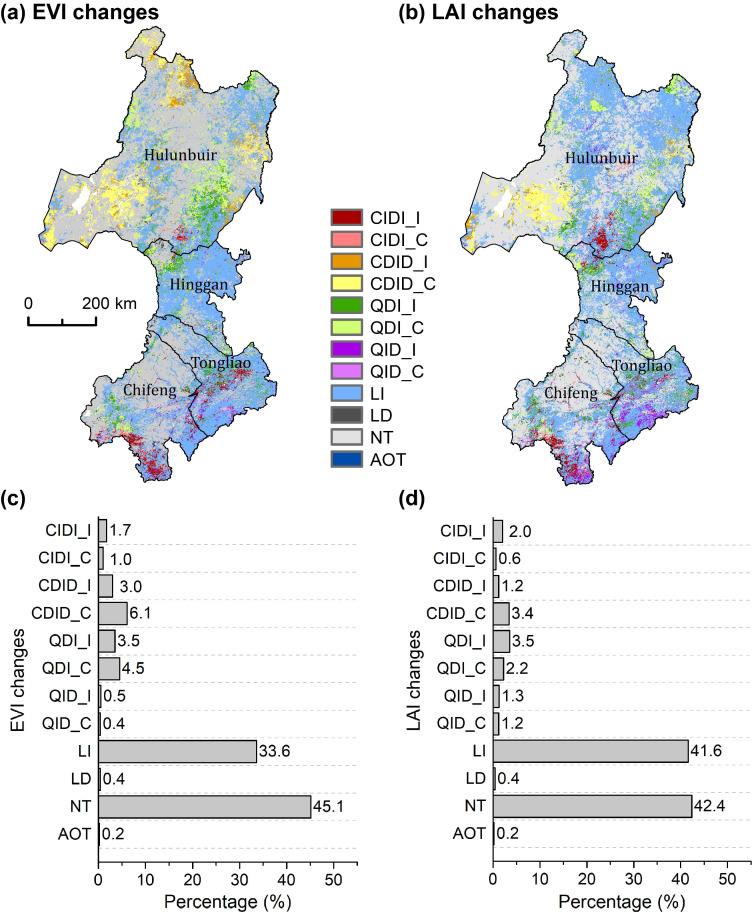
Changes in the growing season mean (**a**) EVI and (**b**) LAI during 2000 to 2016, and the percentages of the types of change in the (**c**) EVI and (**d**) LAI over the study area. CIDI_I represents CIDI change with an increasing overall trend, and CIDI_C represents CIDI change with a non-significant overall trend, i.e., a concealed trend. NT, no trend (*p* > 0.05). AOT, all other types.

Polynomial fitting revealed diverse types of changes in the MODIS EVI, including nonlinear (about 21%), linear (about 34%) and non-significant changes (Figure 2a,c). The major types of nonlinear EVI change included CDID, QDI, and CIDI. Some areas showed a complex spatial pattern of EVI changes. For example, the transition zone between the grasslands and forests in Hulunbuir contained EVI changes of CDID, QDI, and LI. In southern Chifeng, there were spatially adjacent CIDI, QDI, QID, and LI. A more fragmented pattern can be observed in central Tongliao. Moreover, most of the nonlinear changes had concealed or significantly increased overall EVI trends, and a very small proportion of the nonlinear changes showed significantly decreased overall EVI trends. Most linear changes also exhibited an increased EVI. In total, significant EVI decreases were scarce (0.6%), while significant EVI increases were abundant (42.3%).

In general, the spatial pattern of changes in the GEOV2 LAI was similar to that of those in the MODIS EVI (Figure 2b). Major differences occurred in a forest area of northern Hulunbuir, in which a large proportion of the nonlinear changes (CDID) in the EVI were identified as linear changes in LAI. The LAI also exhibited fewer QDI changes in the forest area of southern Hulunbuir. Furthermore, the LAI exhibited more QID changes than the EVI in Chifeng and Tongliao. In total, the LAI showed fewer nonlinear changes (about 16%) than the EVI. In terms of the overall trend, the percentage of significant LAI trends (49.6%) was larger than that of EVI trends. 

### 3.2. Impacts of Climate Variation on EVI and LAI and Residual Trends

**Figure 3:**
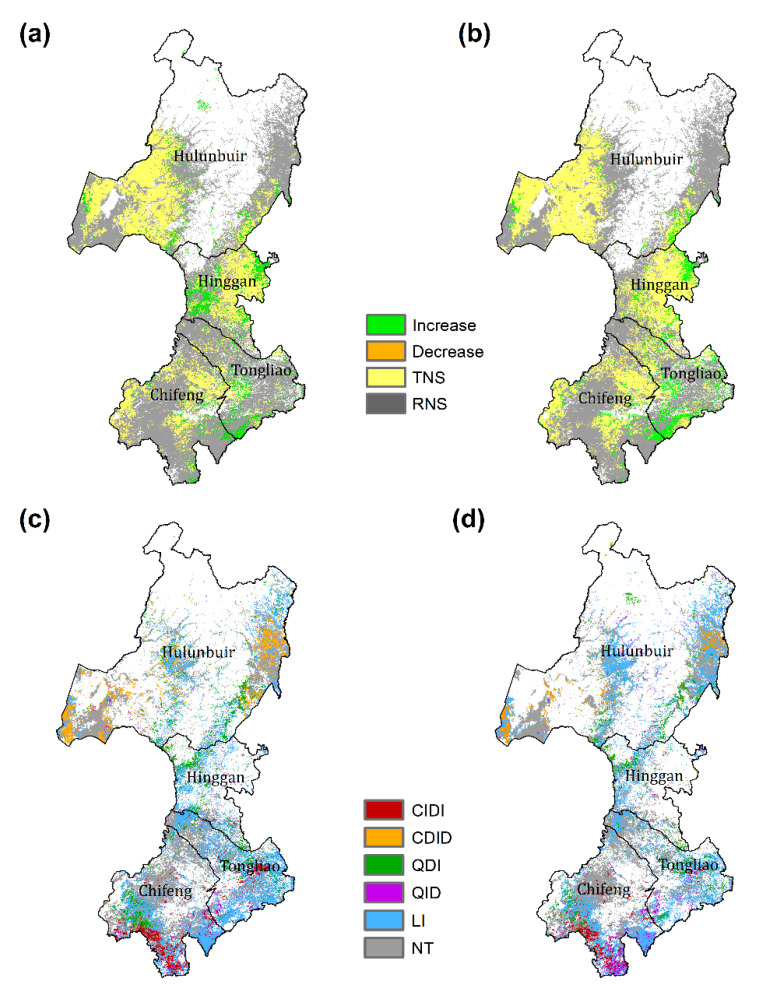
Changes in the residual trends of the (**a**) EVI and (**b**) LAI during 2000 to 2016 for grasslands and rainfed croplands, and the types of change in the (**c**) EVI and (**d**) LAI for the area with non-significant regression between vegetation greenness (EVI and LAI) and climate. TNS, trend in residual was not significant (*p* > 0.05). RNS, regression between vegetation greenness and climate was not significant. NT, no trend (*p* > 0.05).

Linear regression analysis of the relationship between time series of EVI (LAI) and climate and residual trend analysis were performed for only grasslands and rainfed croplands, which account for about 66% of the total pixels. Please note that the following percentages were all computed based on these analyzed pixels. The EVI and LAI showed very similar responses to climate variation (Figure 3). Significant EVI-climate regression accounted for 37.4% of the pixels of grassland and rainfed croplands (Table 2). For the LAI, the percentage of significant regression was a little larger than that of EVI but less than 50%. Furthermore, both the grasslands and rainfed croplands showed weaker climate control pixels than significant climate control pixels (Table 2). These indicate that widespread non-climate factors were largely responsible for the temporal variations in vegetation greenness over this area during recent years.

For the areas significantly controlled by climate variation, residual trends were mostly not significant and positive (Figure 3a,b). A very small proportion (<0.2% for both EVI and LAI) of the area exhibited a negative residual trend, indicating that non-climate induced decreases in vegetation greenness over this region were scare.

The relationship between climate variation and vegetation greenness of major change types in grasslands and rainfed croplands is shown in Table 3. For the CIDI changes, only 12.1% (EVI) were significantly controlled by climate variation. This weak contribution of climate can also be observed for the QDI changes. The CDID, QID, and LI changes showed larger percentages of significant regression than those of the CIDI and QDI. However, all these values are smaller than 50%, except that for the CDID change of the LAI. These results suggest that the effects of non-climate factors were widespread in all change types.

Figure 3c,d presents the spatial patterns of the changes in the EVI and LAI showing weak climate control. For most of the grasslands and rainfed croplands in Hulunbuir and Xinggan, the interannual variations in vegetation greenness were significantly controlled by climate variations. The weak climate control of vegetation generally occurred in the grasslands of west Hulunbuir and the transition zones of forests and grasslands/croplands across Hulunbuir and Xinggan. For these areas, the major change types included LI, CDID, and QDI. However, in Chifeng and Tongliao, we observed large areas showing weak relationships between vegetation greenness and climate. These areas also experienced diverse changes, including LI, CIDI, QDI, and QID.

**Figure 4:**
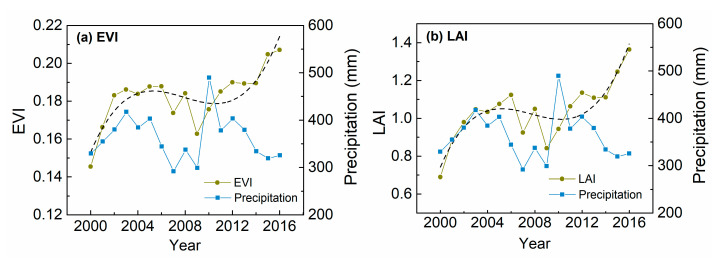
Average (**a**) EVI and (**b**) LAI and precipitation for the area exhibiting CIDI changes in Chifeng.

**Figure 5:**
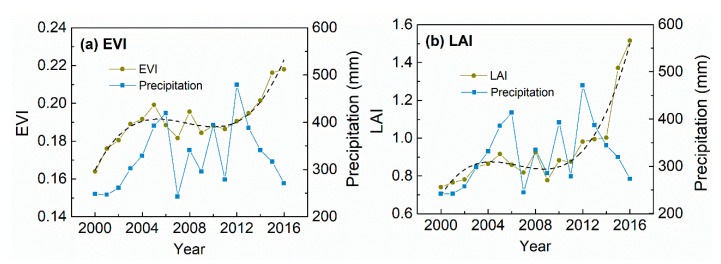
Average (**a**) EVI and (**b**) LAI and precipitation for the area exhibiting CIDI changes in Tongliao.

The average EVI and LAI for the CIDI changes in Chifeng showed a rapid increase at the beginning followed by a slight decrease and then another rapid increase (Figure 4a). As presented in Figure 3, areas with CIDI changes in Chifeng generally exhibited weak responses to climate variation. Although the overall trend cannot be attributed to trends in precipitation, the decreasing phase—which generally occurred during 2006 to 2010—should be a result of a decline in precipitation, especially the droughts in 2007 and 2009. As with Chifeng, the decreasing phases of the CIDI changes in Tongliao were also slight and should be caused by droughts (Figure 5).

**Figure 6:**
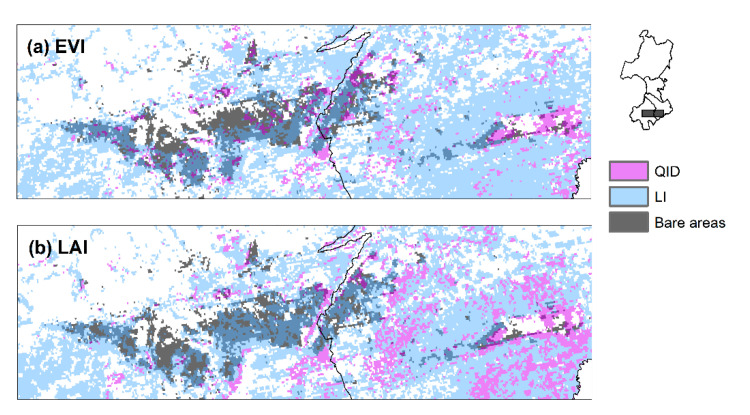
Major changes in (**a**) EVI and (**b**) LAI around the bare areas in the Horqin sandy land.

**Figure 7:**
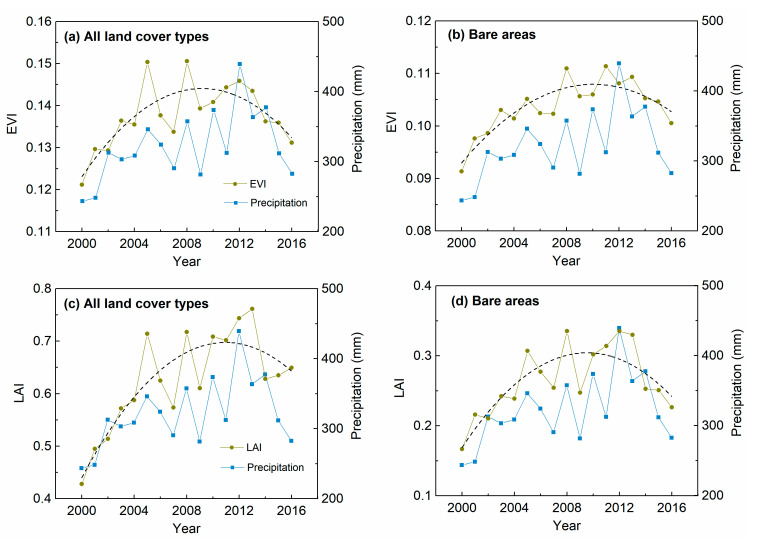
Growing season EVI, LAI, and precipitation within the selected area in the Horqin sandy land. EVI for (**a**) all land cover types and (**b**) bare areas. LAI for (**c**) all land cover types and (**d**) bare areas.

A sub-region showing QID changes around the bare areas in Horqin sandy land was selected. Figure 6 shows the major change types in the EVI and LAI over this area. Changes in both the LAI and EVI were dominated by LI changes, and the spatial distribution of QID was generally adjacent to that of LI, which may imply a local difference of forces. Furthermore, the LAI had more total QID changes than the EVI, while the EVI had more QID changes in bare areas. A comparison of the EVI (LAI) time series with precipitation suggests that the decreasing phases in recent years may be a result of decreased growing season precipitation (Figure 7).

## 4. Discussion

Diverse types of change in the growing season EVI and LAI (2000–2016) were observed in East Inner Mongolia, China, using the polynomial change fitting method. The area exhibited widespread overall increasing trends in both the EVI and LAI. This widespread greening has also been reported in previous studies, e.g., [15,29,37,50,51]. Changes in the EVI and LAI were mainly influenced by factors other than climate for more than half of the grasslands and rainfed croplands. Several previous studies have attributed the greening trends to human activities such as afforestation [9,10], irrigation [15] and fertilization [17]. In this study, nonlinear dryland vegetation changes and their relationship with climate variation were spatially heterogeneous, highlighting the heterogeneity of the drivers of change within specific regions.

Most of the CIDI changes were observed in Chifeng and Tongliao, and were not significantly controlled by climate variation (Table 3). The CIDI changes may reflect different land surface processes under different environmental conditions in Chifeng and Tongliao. In Tongliao, the CIDI changes mainly occurred in the ecotone of croplands and grasslands, while for Chifeng, the CIDI changes were mainly located in the ecotone of forests and grasslands. Tree planting and growth may be the major non-climate factors in CIDI changes in Chifeng. Yin et al. [52] also observed forest gain in this area. For Tongliao, the non-climate drivers of CIDI changes may include efforts to combat desertification and agricultural practices [17]. Although there may exist different non-climate drivers, we observed that the decreasing phases in CIDI changes were mainly caused by droughts for both Chifeng and Tongliao (Figure 4). These further highlight the impacts of droughts on human-induced vegetation greening as discussed in previous studies, e.g., [23,25]. Thus, ecosystem resilience analysis with satellite time series regarding droughts should be a research priority for this region, to determine the ecosystem’s status and risk of degradation [23,26].

For the areas with increasing and non-significant overall trends in the EVI and LAI, the nonlinear analysis showed some decline in both indices in the most recent years (i.e., CDID and QID changes). In particular, some QID changes around the large patch of bare areas in the Horqin sandy land were observed (Figure 6). Positive-negative reversal in gross primary productivity during 2000 to 2016 was also detected in Chifeng and Tongliao by Ma et al. [29]. The decline in vegetation greenness was mainly caused by the decline in precipitation in recent years. Although this region experienced a general greening trend, the observed decline in vegetation greenness may be an early signal of land desertification. Particular attention needs to be paid to tree mortality during recent years for areas with QID changes, as tree planting is one of the major measures employed to combat desertification in the Horqin sandy land [26,53].

Nonlinear analysis with polynomial fitting provides valuable information for understanding dryland dynamics in this region. A limitation is that this fitting method may not precisely capture the turning points. However, we focused more on the types of nonlinear processes in this study, and this tool meets the requirements for this. Furthermore, dryland dynamics were assessed in terms of temporal changes in greenness (EVI and LAI) at a 1 km resolution in this investigation. Some ecologically indicative information may not be reflected in greenness variation [54,55]. Moreover, the drivers of areas with no change in vegetation greenness and that were not significantly controlled by climate were unclear, and need further attention. Higher resolution satellite images, such as those from Landsat and Sentinel, or land surface parameters related to other aspects of ecosystem structure and function, may improve the interpretability of the changes [55,56,57]. Accordingly, to depict dryland processes more comprehensively in this degraded region, further studies may integrate multi-dimensional or multi-scale analyses [56,57].

## 5. Conclusions

We characterized nonlinear changes in dryland vegetation greenness in eastern Inner Mongolia, China from 2000 to 2016 using MODIS EVI and GEOV2 time series. Nonlinear EVI changes occurred for about 21% of the region, and for the LAI, the percentage of nonlinear changes was about 16%. Some areas showed diverse change types, reflecting spatially heterogeneous dryland dynamics in this ecotone. For the overall monotonic trends, very small percentage decreases (less than 1%) and widespread increases in the EVI and LAI were detected.

Most of the grasslands and rainfed croplands exhibited weak responses of vegetation greenness to climate variation. These weak vegetation-climate relationships were found for all the major change types except CDID changes, which were mainly located in the grasslands of Hulunbuir. The CIDI changes showed small percentages of significant response to climate variation, suggesting strong effects of non-climate drivers. For the CIDI changes in Chifeng and Tongliao, the increasing phases may be primarily driven by human activities, whereas droughts induced the decreasing phase.

The QID changes were detected near the bare areas in the Horqin sandy land. The most recent decreasing phase induced by the drying climate highlights the risk of re-degradation, to which attention should be paid to combat desertification. Moreover, the areas with no EVI (LAI) trends and that were not significantly controlled by climate variation also warrant further investigation.

## Figures and Tables

**Table 1:** Types of change in time series characterized by the polynomial change fitting method.

Change Types	Description
CIDI	Cubic, increase–decrease–increase
CDID	Cubic, decrease–increase–decrease
QDI	Quadratic, decrease–increase
QID	Quadratic, increase–decrease
LI	Linear, increase
LD	Linear, decrease

**Table 2:** Percentages of significant regression between climate and vegetation greenness (EVI and LAI) for grasslands and rainfed croplands.

	EVI	LAI
Grasslands	33.9%	39.6%
Rainfed croplands	38.3%	44.0%
All	37.4%	43.0%

**Table 3:** Percentages of significant regression between climate and vegetation greenness (EVI and LAI) for major change types in grasslands and rainfed croplands ^1^.

	EVI	LAI
CIDI	12.1%	10.4%
CDID	48.3%	61.7%
QDI	11.2%	14.8%
QID	35.7%	43.6%
LI	40.4%	39.7%
NT	37.5%	47.9%

^1^ Nonlinear changes with increasing and concealed overall trends were combined, and deceasing trend were not considered.
